# The SULFs, Extracellular Sulfatases for Heparan Sulfate, Promote the Migration of Corneal Epithelial Cells during Wound Repair

**DOI:** 10.1371/journal.pone.0069642

**Published:** 2013-08-08

**Authors:** Inna Maltseva, Matilda Chan, Ina Kalus, Thomas Dierks, Steven D. Rosen

**Affiliations:** 1 Department of Anatomy, University of California San Francisco, San Francisco, California, United States of America; 2 Department of Chemistry, Biochemistry I, Bielefeld University, Bielefeld, Germany; Indiana University School of Medicine, United States of America

## Abstract

Corneal epithelial wound repair involves the migration of epithelial cells to cover the defect followed by the proliferation of the cells to restore thickness. Heparan sulfate proteoglycans (HSPGs) are ubiquitous extracellular molecules that bind to a plethora of growth factors, cytokines, and morphogens and thereby regulate their signaling functions. Ligand binding by HS chains depends on the pattern of four sulfation modifications, one of which is 6-O-sulfation of glucosamine (6OS). SULF1 and SULF2 are highly homologous, extracellular endosulfatases, which post-synthetically edit the sulfation status of HS by removing 6OS from intact chains. The SULFs thereby modulate multiple signaling pathways including the augmentation of Wnt/ß-catenin signaling. We found that wounding of mouse corneal epithelium stimulated SULF1 expression in superficial epithelial cells proximal to the wound edge. *Sulf1^−/−^*, but not *Sulf2^−/−^*, mice, exhibited a marked delay in healing. Furthermore, corneal epithelial cells derived from *Sulf1^−/−^* mice exhibited a reduced rate of migration in repair of a scratched monolayer compared to wild-type cells. In contrast, human primary corneal epithelial cells expressed SULF2, as did a human corneal epithelial cell line (THCE). Knockdown of SULF2 in THCE cells also slowed migration, which was restored by overexpression of either mouse SULF2 or human SULF1. The interchangeability of the two SULFs establishes their capacity for functional redundancy. Knockdown of SULF2 decreased Wnt/ß-catenin signaling in THCE cells. Extracellular antagonists of Wnt signaling reduced migration of THCE cells. However in SULF2- knockdown cells, these antagonists exerted no further effects on migration, consistent with the SULF functioning as an upstream regulator of Wnt signaling. Further understanding of the mechanistic action of the SULFs in promoting corneal repair may lead to new therapeutic approaches for the treatment of corneal injuries.

## Introduction

The corneal epithelium, like other epithelial barriers, encounters physical, chemical, and pathogen insults, often resulting in a wound and a loss of barrier functions. Proper healing of corneal wounds is crucial for maintaining corneal transparency. Healing of the corneal epithelium begins with superficial cells adjacent to the wound migrating as a sheet to resurface the defect [1]–[3]. There is little or no proliferation in corneal epithelial cells until wound closure occurs [4]–[6]. Numerous growth factors, cytokines, morphogens, and ECM proteins, derived either from the epithelium or the underlying stromal layer, have been implicated in the regulation of migration and proliferation of the epithelial cells during corneal repair (reviewed in [2], [7]).

Studies in mice and other model organisms have documented diverse roles for heparan sulfate proteoglycans (HSPGs) in regulating growth factor and morphogen signaling during development and in physiologic/pathophysiologic processes [8]–[13]. HSPGs are comprised of heparan sulfate chains, which are covalently linked to a restricted number of core proteins [13]. HSPGs are associated with almost all animal cells on the cell surface and in the extracellular matrix. HS chains are linear polymers containing repeating disaccharide units of uronic acid and glucosamine, which can be sulfated at N-, 6-O and 3-O positions of glucosamine and 2-O position of uronic acid [14]. HSPGs bind to an enormous number of growth factors, morphogens, cytokines, matrix proteins, enzymes, and cell adhesion molecules. Ligand binding by HSPGs generally depends on the structure of the heparan sulfate chains, in particular the density and pattern of sulfation modifications.

Recently, it has become appreciated that HS chains are post-synthetically modified through the action of two extracellular endosulfatases, SULF1 and SULF2 [15], [16]. The two proteins are highly homologous (63–65% identical in amino acid sequence in mouse and human) and highly conserved in sequence (93–94% identical between species orthologs) and domain organization [16]. The SULFs function at neutral pH to remove 6OS from internal glucosamine residues within highly sulfated subregions (S domains) of intact HSPGs [16]–[18]. Unlike the lysosomal sulfatases which function as “exoenzymes” with activities directed at the non-reducing termini of glycan substrates, the SULFs are endosulfatases in that they act on internal 6OS within intact HS chains [16]–[18].

Through this extracellular remodeling of intact HSPGs, the SULFs impact signaling by a diverse set of growth factors and morphogens (reviewed in [19], [20]). Among the SULF-modulated pathways, Wnt/ß-catenin, GDNF, BMP, FGF-2, TGF-ß1, and PDGF signaling are the most thoroughly investigated [17], [18], [21]–[25]. The SULFs are thought to augment signaling through the ability of the enzyme to liberate ligands from HSPG sequestration. In so doing, the enzyme renders a ligand bioavailable for interaction with its signal transduction machinery. SULF potentiation of Wnt/ß-catenin signaling exemplifies this form of positive regulation [17]. In contrast, the SULFs can antagonize signaling by disrupting the participation of an HSPG as a member of a signaling complex, as occurs in the case of FGF-2 signaling [21].

Not surprisingly, since HSPGs interact with signaling molecules that regulate cell growth and migration, they are implicated in settings of epithelial wound repair. Thus, mice that are null for certain syndecans (cell surface HSPGs) are delayed in wound repair of skin and cornea [26], [27]. Corneal epithelial cells lacking syndecan-1 show reduced migration at the wound edge [26]. In view of the importance of HSPGs in corneal wound repair and the potential for the SULFs to modulate HSPG function, we chose to investigate these enzymes in corneal wound healing.

## Results

### Sulf1 expression increases in wounded mouse cornea

*Sulf1^−/−^* and *Sulf2^−/−^* mice have been generated in a number of labs [22], [28]–[30], but ocular phenotypes have not been reported. Stereo microscope examination of the eyes of *Sulf1^−/−^* and *Sulf2^−/−^* mice showed corneas of normal appearance and size with no evidence of clouding or ulceration. Histological analysis demonstrated no abnormalities in the thickness or morphology of the cornea (Figure S1), indicating the SULFs are dispensable for normal morphogenesis and homeostasis of the cornea. To first explore whether the SULFs might be involved in corneal wound repair, a pilot experiment was carried out to determine the expression of *Sulf* transcripts in needle-scratched mouse corneas. By quantitative PCR, weak *Sulf1* expression was detected in unwounded corneas, which increased by ∼2.5 fold (p = 0.05) in the wounded corneas at 24 hrs (Figure S2), a time at which the wounds had not yet healed. *Sulf2* transcripts were detected in resting cornea but did not change with injury.

**Figure 1:**
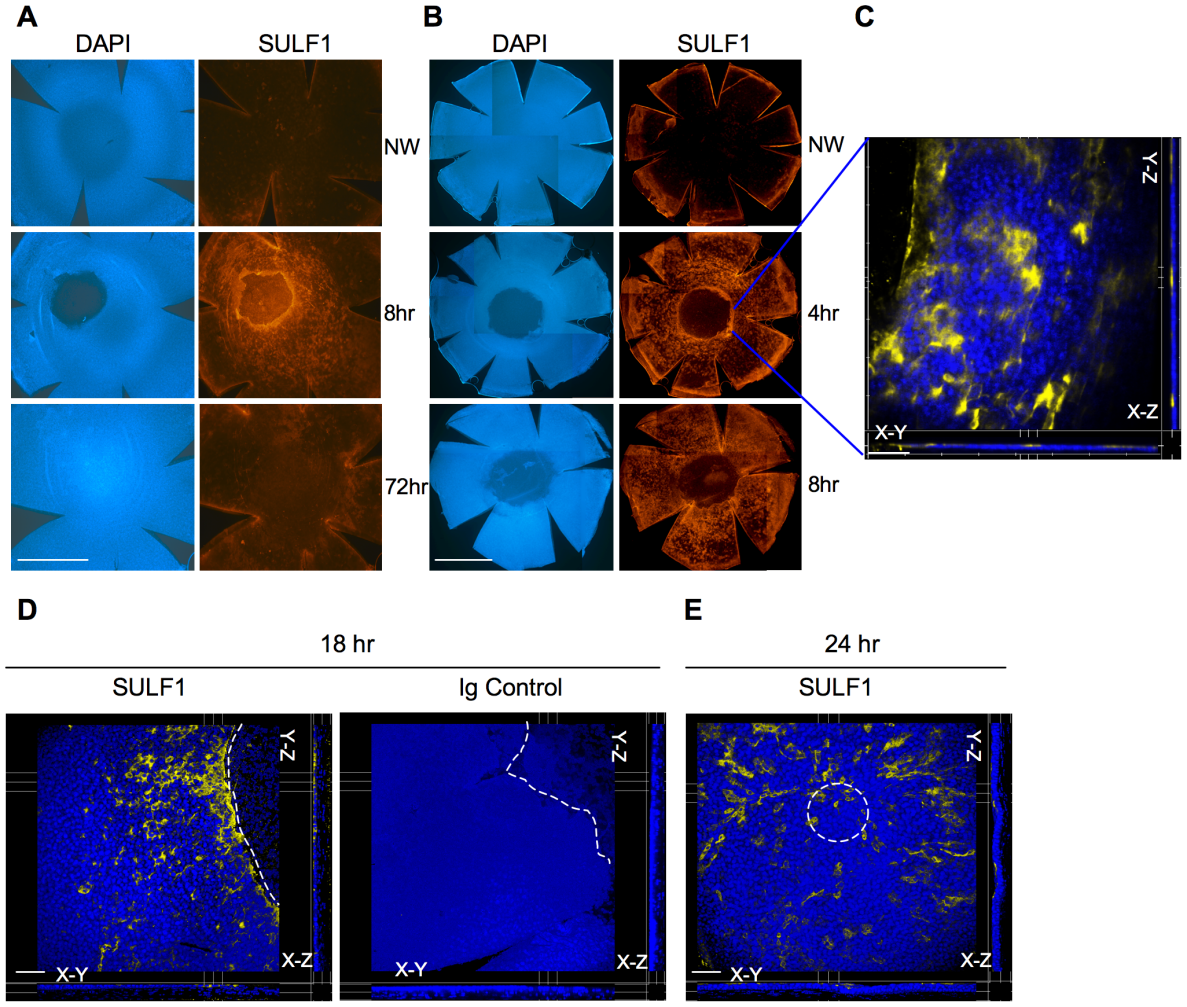
SULF1 expression in injured mouse cornea. **A** and **B:** Immunofluorescent staining with anti SULF1 antibody (R1.1) (right panels, red) and nuclear staining with DAPI (left panels, blue). Corneal wounding of wild-type mice was performed with an Algerbrush. Eyeballs were fixed at 8 and 72 hrs (A) or 4 and 8 hrs (B) post wounding, corneas were prepared for whole mount staining, and imaged at 4× by immunofluorescent microscopy (composite images are prepared digitally). A contralateral eye served as a non-wounded control (NW). The pair in each row shows the same field. Scale bar: 1 mm. **C:** Imaging of the 4-hr post-injury corneal whole mount at 40× by spinning disc confocal microscopy. The field shown is centered on the wound edge. SULF1 (yellow) is only found in the superficial epithelial layer (X-Z and Y-Z panels). Scale bar: 70 µm. **D:** Whole mounts of 18 hr post-wounded corneas stained with either anti SULF1 antibody (R1.1) or rabbit anti-DNP (IgG control) and imaged by laser scanning microscopy at 20×. The wound margin is visible in the upper right-hand side corner and outlined by the dotted white line. **E:** Whole mount of cornea 24 hrs post-wounding stained with R1.1 and imaged by laser scanning microscopy at 20×. The dotted white line approximates the center of the original wound. Scale bars: 100 µm.

To extend these preliminary findings and look for protein expression, we switched to a more controlled and quantifiable method for corneal epithelial debridement by employing a pressure-controlled electric brush (Algerbrush), which removes a circular patch of epithelial cells, leaving the basement membrane intact [31]. This instrument was used to produce circular 1 mm wounds, which were positioned in the central cornea. An affinity-purified peptide-specific antibody (R1.1) was employed, which reacts with SULF1 but not SULF2 (Figure S3). Examination of whole mount preparations revealed a very scant, dispersed expression of SULF1 in uninjured cornea (Figure 1A). 8 hrs after the generation of the wound, there was a strong positive signal, which was concentrated primarily in cells at the wound edge (Figure 1A). Distal to the wound, SULF1 was present in a scattered pattern, which resembled its distribution in unwounded cornea. By 72 hrs when the wound was completely healed by fluorescein staining (which detects an incomplete epithelial barrier) and histological analysis (not shown), there was no SULF1 signal above the baseline pattern (Figure 1A). In a separate experiment, there was very strong staining at the wound edge 4 hrs after injury, which was comparable to that at 8 hrs. When the cornea was visualized at 4 hrs by confocal microscopy, the SULF1 expression around the wound was confined to the superficial epithelium with no staining of the basal epithelium or stroma (Figure 1C, X–Z, Y–Z planes). 18 hrs after wounding, stained cells remained concentrated close to the wound margin in the superficial layer (Figure 1D). At 24 hrs, when the wound was ≈95% closed by fluorescein staining, positively stained cells were oriented around the center of original wound (Figure 1E). Additionally, immunohistochemistry performed with three other SULF1 antibodies revealed the same pattern of SULF1 induction (see Figure S4 for staining with one of these). There was no staining of wounded cornea when isotype control antibodies were used (Figure 1D, Figure S4). To evaluate SULF2 expression, we used three antibodies (2B4, 2.1 and 2.3), which have been validated for immunocytochemistry [24], [25], [32], [33]. SULF2 was not detectable in either unperturbed or injured corneal epithelium (not shown).

### Sulf1 deficiency delays corneal re-epithelialization

**Figure 2:**
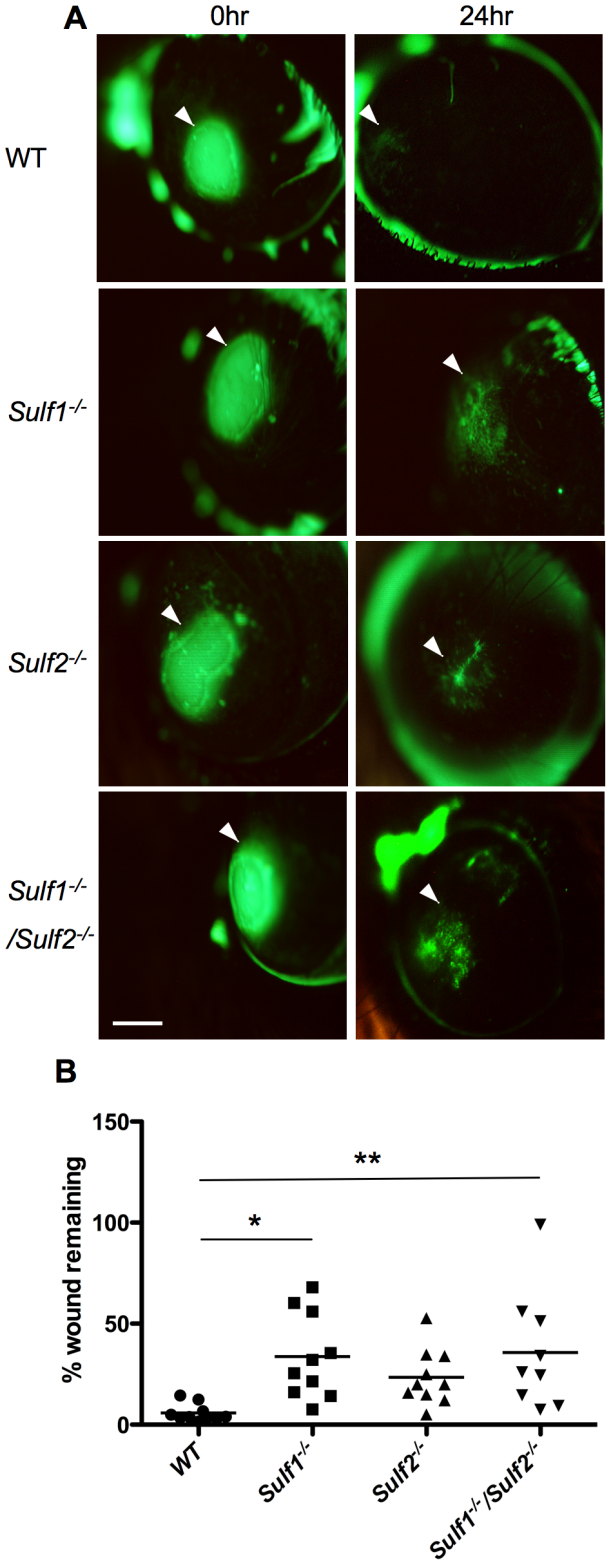
SULF1-dependent corneal re-epithelialization in vivo after wounding. **A:** Epithelial defects (∼1 mm diameter) created at the center of wild-type (WT), *Sulf1* ^−/−^, *Sulf2* ^−/−^, and *Sulf1* ^−/−^/*Sulf2* ^−/−^ corneas (left panel) were visualized by fluorescent staining (arrows) immediately post-wounding (left panel) and 24 hrs after wounding (right panel) with individual mice used for each condition. Representative results are shown for each genotype at each time point. Scale bar: 1 mm. **B:** The two-dimensional projection of the wound area was quantified using ImageJ software, and the percent remaining epithelial defect was determined (Area _t24_/Area _t0_×100) (+ SEM, 11–14 eyes/genotype; *p<0.05, **p<0.01 compared to WT). Statistical significance was evaluated by one-way analysis of variance (ANOVA) with a Tukey's range post-test. *P* values of <0.05 were considered significant.

The upregulation of SULF1 in the injured epithelium suggested a possible role for this protein during epithelial healing. We therefore investigated the healing of corneal epithelial wounds in *Sulf1^−/−^, Sulf2^−/−^, and Sulf1^−/−^/Sulf2^−/−^* mice. Wounds were generated as above and photographed immediately and 24 hrs later. Wound areas were measured by fluorescein staining (Figure 2A). Wild-type mice showed almost complete healing by 24 hrs (5.8%±1.3 of starting wound area, n = 11), whereas substantial wounds were present in the *Sulf1^−/−^* mice (33%±6.1, n = 11) and *Sulf1* ^−/−^/*Sulf2* ^−/−^ mice (38%±8.7, n = 10) (Figure 2B). Healing in *Sulf2* ^−/−^ mice (19.1%±4.1, n = 14) showed a trend to be delayed that did not attain statistical significance. As determined by stereo-microscope examination and fluorescein staining, wounds in the mutant mice (*Sulf1^−/−^*) eventually healed (96 hrs) with the cornea returning to a normal histology (not shown). We conclude that SULF1 is required for optimal re-epithelialization of wounded cornea.

### Sulf1^−/−^ primary corneal epithelial cells migrate slower in a scratch-wounded monolayer

**Figure 3:**
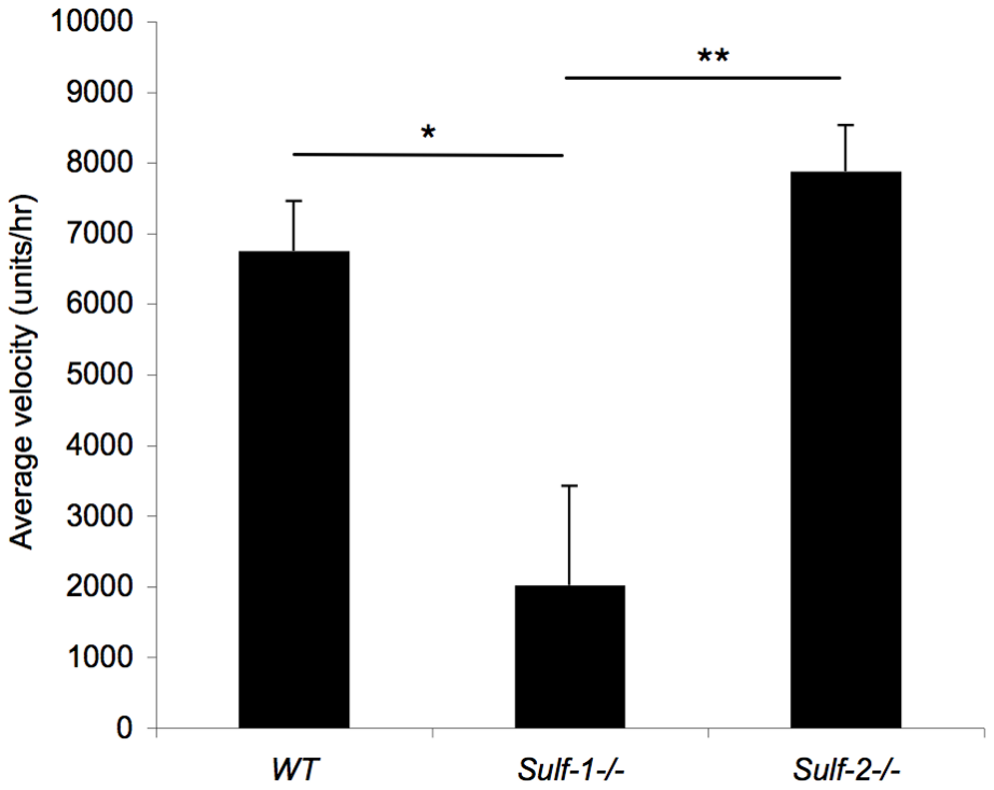
In vitro wound closure in primary cultures of mouse corneal epithelial cells (MCE). Confluent monolayers of MCE cells of the indicated genotype were wounded by a pipette tip and monitored by time-lapse microscopy for 24 hrs. Wound healing was determined as the average linear speed of the wound edge over 8 hrs. *Sulf1^−/−^* MCE migrated at a reduced rate compared to wild-type (WT) and *Sulf2^−/−^* cells. Data are expressed as means+SEM, n = 3 per group. Statistical significance was evaluated with a Student t-test. *p = 0.023, **p = 0.009.

The upregulation of SULF1 in the superficial cells of the injured cornea led us to focus on the potential role of SULF1 in regulating cell migration during wound repair. This was first investigated in scratch-wounded monolayers of primary mouse corneal epithelial cultures. Corneal epithelial cells were isolated and cultured by published procedures [34]. Cells from *Sulf1^−/−^*, and *Sulf2^−/−^* mice, like those from wild-type mice, formed confluent monolayers with a cobblestone morphology, a characteristic organization of epithelial cells. Upon scratch wounding of confluent monolayers, cells at the margins of the wound and their neighbors became migratory and closed the gaps. *Sulf1* ^−/−^ corneal epithelial cells migrated at a greatly reduced rate (2022±1408 units/hr) compared to wild-type cells (6758±700 units/hr, p = 0.023) and *Sulf2* ^−/−^ cells (7877±655 units/hr, p = 0.009) (Movies S1, S2, S3, Figure 3). The speed of migration was calculated based on the 8 hr interval, a time frame that excludes a significant contribution of cell division to gap closure, since the doubling time of these cells is ≈24 hr.

### SULF2 knockdown in cultured human corneal epithelial cells slows migration

**Figure 4:**
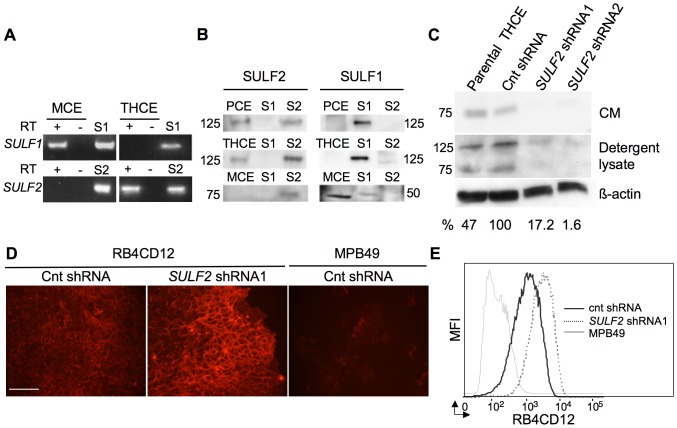
SULF expression in corneal epithelial cells. **A:** RT-PCR analysis for *SULF* expression was performed on cDNA prepared from THCE, MCE and 293T cells transfected with pcDNA *Sulf1* (S1) or pcDNA *Sulf2* (S2) as positive controls. First-strand cDNA synthesis was also performed with reverse transcriptase omitted as a control for genomic DNA contamination (RT-). **B:** Detergent lysates of human primary corneal epithelial cells (PCE), THCE, MCE and pcDNA *SULF1* (S1) or pcDNA *SULF2*-transfected 293T (S2) cells were subjected to Western blot analysis with 1A4 (SULF1) and R2.1/2.3 (SULF2). SULF1 was detected in MCE cells by the presence of its C-terminal subunit (∼50 kDa), while SULF2 was absent. SULF2 was detected in THCE and PCE cells (as the 125 kDa proprotein). **C:** SULF2 in the indicated THCE cells. Western blotting for SULF2 (R2.1/R2.3) was performed on conditioned media (CM) and detergent lysates from these cells. Density of each band was measured with ImageJ, values were normalized to β-actin of the Cnt shRNA-treated cells, and the percent SULF2 expression (shown at the bottom of the respective lanes) was calculated relative to the expression of the protein in Cnt shRNA cells. shRNA1 and shRNA2 reduced SULF2 expression by ∼83% and ∼98% respectively. **D:** and **E:** Effects of SULF2 knockdown on the cell-surface sulfation was determined by staining with RB4CD12. **D:** Immunofluorescent staining of RB4CD12 or control MPB49 antibody in THCE control- and SULF2-knockdown cells. **E:** Flow cytometry analysis of RB4CD12 or control MPB49 staining in THCE control- and SULF2-knockdown cells. SULF2 knockdown increased the level of RB4CD12 epitope on the cell surface by 72±2% (mean ± SEM, n = 3 experiments). A representative result is shown.

To manipulate SULF levels in corneal epithelial cells, we turned to a human corneal epithelial cell line (THCE). Surprisingly, *SULF2* but not *SULF1* transcripts were detected in these cells (Figure 4A). Correspondingly, SULF2 but not SULF1 protein was present in detergent lysates of the cells, as was also the case for human primary corneal epithelial cells (PCE) (Figure 4B). Consistent with the immunocytochemistry on mouse cornea described above, primary mouse corneal epithelial cells (MCE) expressed only SULF1 (Figure 4B). These findings indicate that corneal epithelial cells of mouse and human have undergone a switch between SULF1 and SULF2.

**Figure 5:**
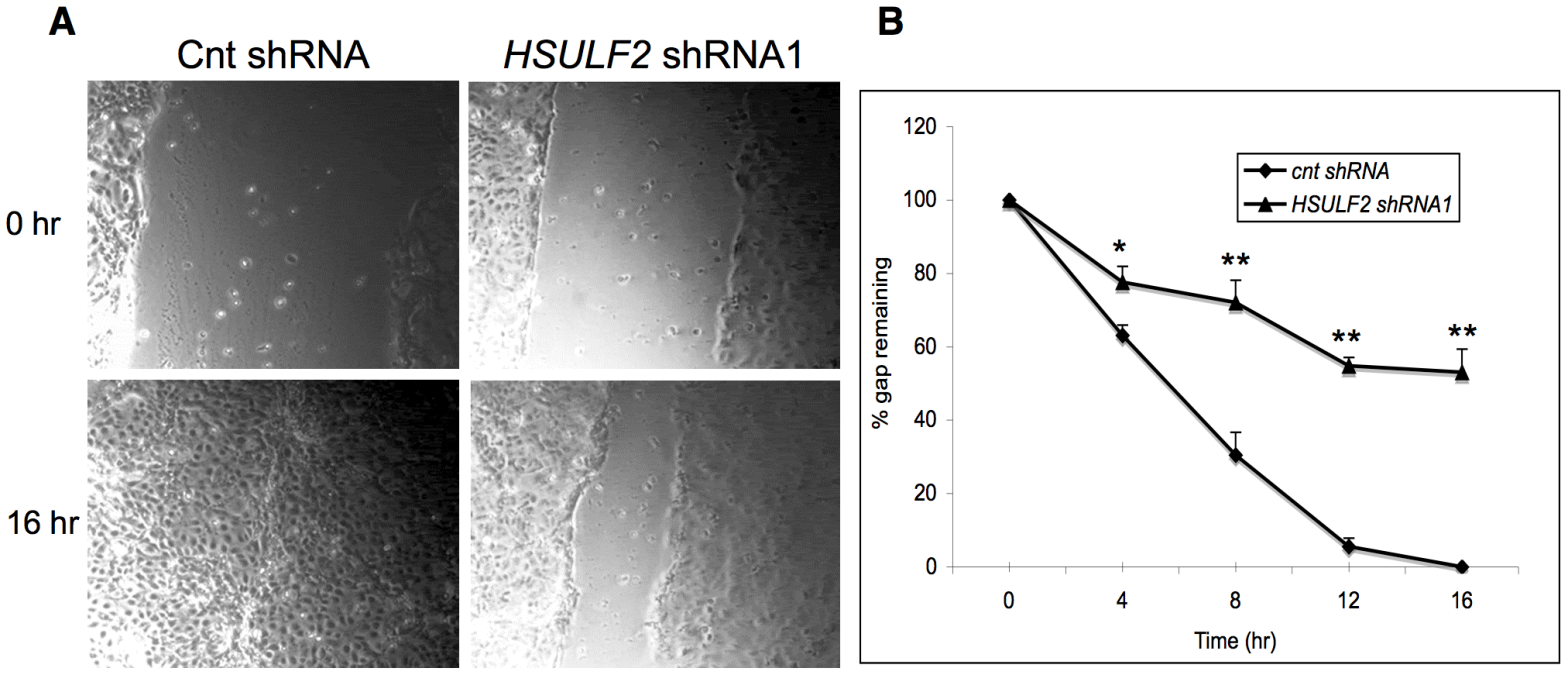
Impaired migration in SULF2-knockdown THCE cells. Confluent monolayers were wounded by a linear scratch, and wounds were monitored by time-lapse microscopy. **A:** Representative phase-contrast images of the gap after 0 and 16 hrs in mock-transformed (Cnt shRNA) and SULF2-knockdown cells (*HSULF2* shRNA1). **B:** Percent gap remaining at the indicated times was determined. Data are expressed as means+SEMs, n = 3 replicate wells per group. ** p<0.01, *p<0.05, Student t-test.

To investigate the contribution of SULF2 to THCE migration, we employed a previously validated lentivirus shRNA methodology to achieve knockdown of the protein [24], [25], [33]. THCE cells were transduced with either of two SULF2 shRNAs or a control shRNA. SULF2 was reduced by 83% (pLV-1413, shRNA1) and by 98% (pLV-1143, shRNA2) in whole cell lysates with normalization to β-actin (Figure 4C). Comparable knockdowns were achieved in conditioned medium derived from the cells (Figure 4C). To determine whether the enzymatic activity of SULF2 was correspondingly suppressed, we employed a phage display antibody (RB4CD12), whose binding to HSPGs requires the 6OS modification within highly sulfated subdomains [35]. This antibody has been validated as a reporter for SULF enzymatic activity [24], [25], [36]. By immunofluorescence and flow cytometry, RB4CD12 stained THCE cells, whereas a non-HS-reactive antibody MPB49 bound at the background level (Figure 4D, 4E). As predicted, there was increased RB4CD12 staining of THCE cells with SULF2 knockdown (Figure 4D). By flow cytometry, Mean Fluorescence Intensity (MFI) increased by 72±2% in *SULF2* shRNA-transduced cells compared to control shRNA transduced cells (Figure 4E). With confirmation of reduced enzymatic activity in the knockdown cells, the contribution of SULF2 to THCE cell migration was investigated. As shown in Figure 5 and Movies S4, S5, knockdown cells exhibited slower wound closure at all time points (4, 8, 12, and 16 hrs). For example, at 16 hrs, 53±6.3% of the gap remained in the knockdown cells, whereas the control shRNA-transduced cells had completely closed the gap (p = 0.0007). Non-transduced cells behaved like the control shRNA-transduced cells (data not shown). To investigate the possible contribution of SULF2 to cell proliferation, bromo-deoxyuridine (BrdU) incorporation was compared in these cell populations. There was no difference between control (35.2%±2.7) and SULF2 knockdown THCE cells (31.7%±2.3, p = 0.157, Figure S5).

**Figure 6:**
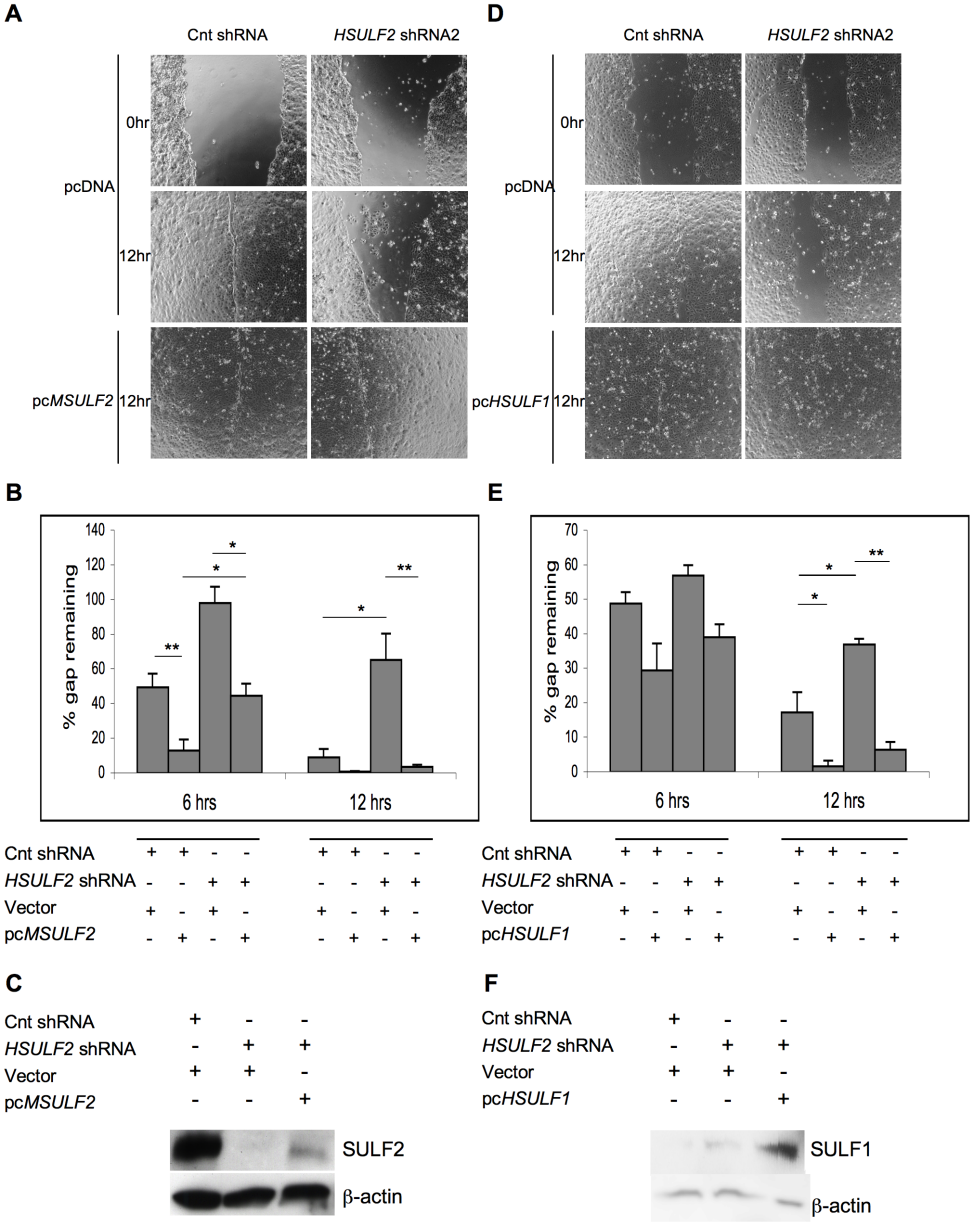
Rescue of migration defect in SULF2 knockdown THCE cells by MSULF2 or HSULF1 overexpression. **A:** and **D:** Representative phase-contrast images of the gap at 0 and 12 hr post-scratch in mock-transfected (Cnt shRNA) and SULF2-knockdown cells (*HSULF2* shRNA2) transfected with pcDNA or pc*MSulf2* (A) or with pcDNA or pc*HSulf1* (C). **B:** and **E:** Percent gap remaining at 6 and 12 hrs was determined for the indicated conditions. Data are expressed as mean+SEM, n = 4 replicate wells per treatment. **p<0.01, *p<0.05, Student t-test. **C** and **F:** Overexpression of SULF in SULF2 knockdown THCE cells by plasmid transfection. SULF was detected in lysates of the indicated THCE cells by Western blotting with R2.1/2.3 for SULF2, 75 kDa subunit (C) and G1.6 for SULF1, 75 kDa subunit (F). β-actin was used as a loading control. A high level of endogenous SULF2 was observed in Cnt shRNA-treated cells (C, Lane 1), while HSULF2 shRNA almost completely eliminated protein expression (Lane 2). Transient expression with pcMSULF2 in SULF2 knockdown cells partially restored SULF2 expression but below the endogenous level in Cnt shRNA (Lane 3). SULF1 expression was detected only transient transfection with pcHSULF1 (F, Lane 3).

To control for off-target effects from *SULF2* shRNA transduction, we carried out rescue experiments. Transient expression of mouse SULF2 (not targeted by the *HSULF2* shRNA) in cells transduced with the *HSULF2* shRNA accelerated gap closure relative to the same cells transfected with a control plasmid (Figure 6A, 6B). Enhancement was observed at both 6 and 12 hrs after scratching. For example, at 12 hrs, overexpression of SULF2 in knockdown cells resulted in 3.4±1.2% gap remaining vs. 65.0±15.0% in the knockdown cells (p = 0.006). Forced expression of mouse SULF2 in control cells (i.e., control shRNA-treated) accelerated gap closure at 6 hrs (12.8±6.3% vs. 49.1±8.1%, p = 0.012). Mouse SULF2 expression was confirmed by immunoblotting (Figure 6C).

Considerable *in vitro* and *in vivo* evidence has argued for functional redundancy between SULF1 and SULF2 [22], [28], [29], [37]. To directly test for redundant functions in THCE cells, we determined whether SULF1 was able to rescue the migration defect in SULF2 knockdown THCE cells. Transient expression of HSULF1 (not targeted by the *SULF2* shRNA) accelerated gap closure in both control cells and SULF2 knockdown cells (Figure 6E). SULF1 expression was confirmed by immunoblotting (Figure 6F).

### SULF regulation of Wnt/ß-catenin signaling in corneal epithelial cells

**Figure 7:**
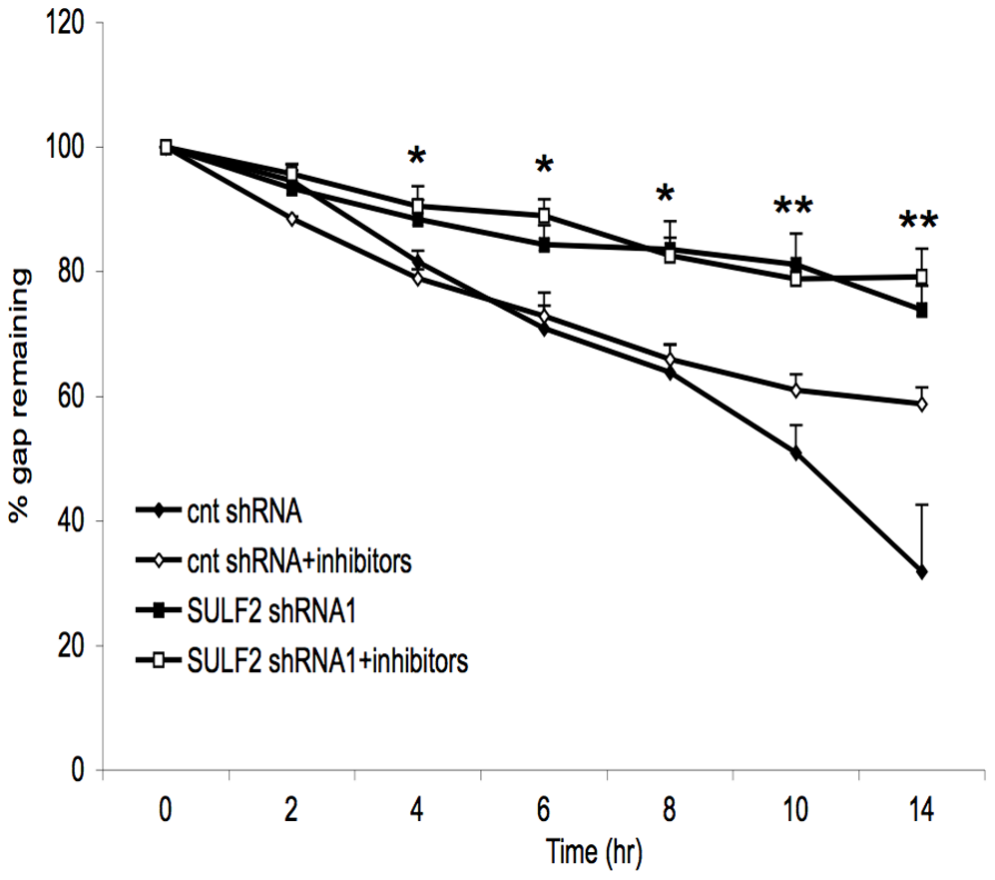
Effects of Wnt inhibition on migration of corneal epithelial cells. Confluent cultures mock- and *SULF2* shRNA transduced THCE cells were wounded and treated with Wnt inhibitors (cocktail of DKK1/sFRP-1) or medium alone. Gaps were quantified at different times and reported as a percent of the remaining gap. Data are expressed as mean + SEM, n = 3 replicate wells per group. Statistical differences were calculated by the Student t-text. * denotes p<0.05, ** denotes p<0.01 for comparison of cells with mock knockdown (cnt shRNA) vs. SULF2 knockdown cells without Wnt inhibitors (SULF2 shRNA1), as well as with added Wnt inhibitors (SULF2 shRNA1+inhibitors). The statistical significance of the other comparisons is given in **Results**.

Wnt signaling has been implicated as an important signaling pathway in THCE cell migration and corneal repair [38], [39]. As the SULFs are established to promote Wnt/ß-catenin signaling in multiple developmental and cancer contexts [17], [24], [33], [40], [41], [42], we sought to determine whether SULF2 regulates Wnt signaling in THCE cells. To measure Wnt/ß-catenin signaling (also known as canonical Wnt signaling), we employed the TOP/FOP flash assay, which quantifies ß-catenin dependent transcriptional activity [43]. As shown in Table 1, TOP/FOP activity was present in THCE cells. shRNA-mediated knockdown of SULF2 reduced TOP/FOP activity to 69±5% of that in control shRNA-transduced cells in 4 independent experiments (p = 0.007). To study the relationship between SULF2-regulated Wnt signaling and cell migration, we determined the effects of extracellular Wnt inhibitors (DKK1 and sFRP-1) on the migration of THCE cells with or without SULF2 knockdown. DKK1 antagonizes Wnt/ß-catenin signaling by interacting with co-receptors (LRP5/6) of Wnt proteins, whereas sFRP-1 neutralizes Wnt ligands by directly binding to them [42], [43]. Consistent with Figure 6B, SULF2 knockdown slowed the migration of THCE in the wounded monolayer assay at all time points (Figure 7). Combined application of DKK1 and sFRP to mock-knockdown THCE cells slowed the rate of gap closure at 14 hrs (58.5±5% with inhibitors vs. 32±21% without inhibitors, p = 0.049) and produced a strong trend for slowing at 10 hrs (p = 0.09). Notably, the effect of SULF2 knockdown in slowing gap closure exceeded that of the Wnt inhibitors, beginning at 4 hrs (p = 0.03) and continuing for all subsequent times. Importantly, gap closure by SULF2 knockdown cells was slowed to the same extent in the presence or absence of the Wnt inhibitors at all time points.

**Table 1:** Effect of SULF2 knockdown on Wnt/ß-catenin signaling in THCE cells.

Experiment	Control shRNA	SULF2 shRNA	% Control
1	26±3	16±4	61.5%
2	6±1	4±0.5	66.6%
3	45±15	29±7	64.4%
4	17±1.6	14±1.4	83.3%
			68.9±4.9%

THCE cells were transduced with either control shRNA or SULF2 shRNA1. TOP/FOP was determined for each cell type in 4 independent experiments as described in **Materials and Methods**. Mean values ±SEMs based on 3 replicate determinations are shown. The TOP/FOP value for control shRNA cells was normalized to 100% within each experiment, and the TOP/FOP signal for the SULF2 shRNA cells was computed relative to this normalized value (% Control). The mean ± SEM is shown (at the bottom right) for the 4 experiments. The normalized values differed between the control shRNA and SULF2 shRNA transduced cells with p = 0.007 by a two-tailed, paired Student t-test.

## Discussion

The SULFs have been studied extensively in development (reviewed in [19], [20]), adult physiology [44], [45], and cancer (reviewed in [20]). Only a few studies have been pertinent to tissue repair. Thus, Roy et al. [46] observed a 40-fold upregulation of *SULF1* in blood vessels associated with wounded skin in human. Langsdorf et al. [47] found upregulation of SULF1 and SULF2 in regenerating muscle fibers of adult mice after a chemical injury. *Sulf1^−/−^/Sulf2^−/−^* mice exhibited delayed repair but there was no defect in single null mice. The defect was exerted at the level of reduced satellite cell differentiation. Later during muscle generation, the SULFs promote myoblast fusion into myofibers [42]. Impairment of corneal wound repair in syndecan-1 deficient mice [26], [27] prompted us to investigate the SULFs in this system. We found that inactivation of *Sulf1* alone was sufficient to produce a marked delay in re-epithelialization of wounded cornea, in contrast to the regeneration of skeletal muscle where the two SULFs appear to function redundantly [47]. The finding that SULF1 was induced only in the superficial cells proximal to the wound suggested that the protein might be involved in regulating cell migration. In support of such a role, we observed that primary mouse *Sulf1^−/−^* MCE cells migrated slower than wild type cells in a scratch-wounded monolayer, a widely used assay for cell migration. Correspondingly, knockdown of SULF2 in THCE cells resulted in slower migration in the same assay. Moreover, the addition of exogenous SULF1 or SULF2 to control or SULF2 knockdown THCE cells accelerated gap closure, providing “gain of function” evidence for SULF regulation of cell migration. It should be noted that wound closure in injured cornea *in vivo* and in the wounded monolayer culture *in vitro* still occurred in the absence of the SULF, indicating that the enzyme facilitates migration rather than being obligatory. Thus, as in muscle regeneration [42], [47], the SULFs appear to function in a fine-tuning capacity.

The SULFs have previously been implicated in cell migration during development and in cancer. Thus, SULF1 knockdown in oligodendrocyte progenitor cells reduced the dispersion of these cells throughout the spinal cord in the rat embryo [48]. Knockdown of both SULFs in *Xenopus* embryos resulted in impaired migration of neural crest cells [49]. In carcinoma cell lines, overexpression of SULF2 enhanced scratch-wound repair of monolayer cultures, whereas knockdown of endogenous SULF2 slowed the closing of scratch wounds in monolayers [24], [50]. Notably, several of the settings in which the SULFs modulate cell migration can be classified as examples of epithelial-to-mesenchymal transition (EMT) [51]. EMT is a complex program of cellular and molecular events in which cells in an epithelium lose or loosen their connections with their neighbors, become motile, and migrate away from the epithelium [51], [52]. A classic morphogenetic example of EMT is provided by neural crest cells, which delaminate from the dorsal neural epithelium and migrate long distances before assuming a variety of fates [52]. Carcinoma cells acquire the ability to invade and metastasize by abnormally activating an EMT program [53]. In wound repair of epidermis and cornea, the behavior of cells at the margin of the wound, (i.e., activation of migration and reduction of intercellular adhesion) recapitulates molecular and cellular features of EMT [52], [54]–[56]. In view of the diverse examples of EMT in which SULFs regulate migratory behavior, these enzymes may emerge as a common feature of EMT programs in development and disease. In support of this possibility, *SULF2* was identified in an unbiased screen for upregulated genes in EMT transitions of cancer cells [57].

A question of major interest has been whether the SULFs are functionally redundant. The initial characterization of the two human SULFs revealed indistinguishable 6OS endosulfatase specificities against heparin [16], [58]. A subsequent study with HS chains as the substrate also supports equivalent endosulfatase activities [58]. However, comparison of heparan sulfate chains obtained from SULF deficient fibroblasts has suggested slightly different specificities for the two enzymes [37]. Numerous studies have documented that mice null for both SULFs exhibit much more pronounced phenotypes than mice deficient in either enzyme alone [22], [28], [30], [41], [45], [47], [59]. The more severe phenotypes of the double null mice is consistent with functional redundancy of the two SULFs whereby each enzyme has the ability to compensate for the absence of the other in critical tissues [22], [28], [47], [59]. The present study provides strong support for the functional interchangeability of the SULFs. Whereas SULF1 is expressed and promotes wound healing of the mouse cornea, there is a switch to SULF2 in primary human corneal epithelial cells, as well as in a human corneal epithelial cell line (THCE). Furthermore, we found that the delay in closing a scratch wound in THCE cells after SULF2 knockdown was rescued by expression of SULF1. Rescue experiments in studies of neural crest migration have led to the same conclusion about the functional redundancy of the two enzymes [49]. It should be noted, however, that a non-overlapping expression of *Sulf1* and *Sulf2* has been documented, in particular in the nervous system [29], [60]. Moreover, specific impairments in the development and maintenance of the brain have been observed in *Sulf1^−/−^* and *Sulf2^−/−^* mice [60].

The signaling pathways modulated by the SULFs during corneal wound repair remain to be fully elucidated. As reviewed above, Wnt/β-catenin signaling is known to be positively regulated by the SULFs in various contexts. In line with the observation that an exogenously provided Wnt ligand (Wnt7a) promotes scratch wound repair of THCE cells [38], the present study demonstrates Wnt/ß-catenin signaling in these cells with the TOP/FOP flash assay. THCE cells are capable of autocrine Wnt signaling (i.e. responsive to their own Wnt ligands), since exogenous Wnt ligands do not have to be added to achieve a TOP/FOP signal. SULF2 knockdown reduced Wnt/ß-catenin signaling in THCE cells by ≈30% (Table 1), which compares to 50–70% inhibition in pancreatic and lung cancer cell lines using the same methodology [24], [33]. Presumably, this dependency is based on SULF-mediated mobilization of Wnt ligands from HSPGs of THCE cells, as has been shown in other systems [17], [41]. The application of extracellular Wnt pathway inhibitors to SULF2 knockdown cells did not further slow the migration of the cells, consistent with SULF2 functioning upstream of Wnt signaling to regulate cell migration. Downstream of the engagement of Wnt cell surface receptors are multiple effector mechanisms that could potentially promote cell migratory behavior, including repression of E-cadherin transcription and the induction of MMPs and various motility factors [61], [62], [63].

Work from several laboratories has provided evidence for the importance of HB-EGF (heparin-binding epidermal growth factor) and its activation of the EGF receptor (EGFR) in wound repair of corneal epithelium [7], [55], [64], [65]. Strikingly, pharmacologic inhibition of either HB-EGF or EGFR signaling slows wound closure in organ-cultured cornea and has parallel effects on the migration of THCE cells in scratch-wounded monolayers [64]. Possible connections between the Wnt pathway and EGFR signaling injury exist in that 1) EFGR is known to be a Wnt target in certain cells [66] and 2) autocrine Wnt signaling can transactivate EGFR signaling in some settings [67]. Alternatively, HB-EGF as a heparan sulfate-binding component could potentially be directly mobilized from HSPG sequestration through SULF action. This direct mobilization pathway could occur in parallel with activation of Wnt signaling, which could explain why SULF knockdown had a greater effect than the extracellular Wnt inhibitors on THCE cell migration (Figure 7). Notably, there is a precedent for SULF modulation of EGFR signaling in that SULF2 knockdown in astrocytoma cells led to a reduction in EGFR activation, as well as that of several other receptor tyrosine kinases [25].

It should be noted that a number of other growth factors implicated in corneal wound closure and/or corneal cell migration (IL-1ß, TNF-α, TGF-α, TGF-ß1, HGF, and KGF) [7], [68], [69] are able to bind heparin/HSPGs (reviewed in [11]) and are thus potentially subject to direct mobilization by SULFs. Because of their proximal position in signaling, the SULFs can influence the activity of more than one HSPG-binding growth factor and thus have pleotropic downstream effects, as has been demonstrated in glioma cells [25].

Corneal injuries due to infections, trauma, thermal and chemical insults represent a serious medical condition that can lead to loss of sight [70]. Diabetics are a particularly vulnerable population at risk for persistent defects [71], [72]. Topical application of EGF has been shown to enhance epithelial corneal repair and reduced healing times in animal experiments and human trials [7]. Since the SULFs act extracellularly, these enzymes could potentially be used for topical application to injured corneas to facilitate epithelial repair.

## Materials and Methods

### Ethics Statement

All experiments were performed in strict accordance with the recommendations in the Guide for the Care and Use of Laboratory Animals of the National Institutes of Health and in strict accordance with the ARVO Statement for the Use of Animals in Ophthalmic and Vision Research. The study was approved by the Institutional Animal Care and Use Committee of University of California of San Francisco (IACUC) with approval number AN085643. Mice were maintained under pathogen-free conditions in the UCSF barrier facility. Mice were anesthetized by using 125 mg/kg 2,2,2-tribromoethanol (Avertin; Sigma-Aldrich, St. Louis, MO) intraperitoneally and by topical 0.5% Proparacaine (Akorn, Buffalo Grove, IL). After wounding, topical 0.5% Proparacaine was placed again on the cornea for post-procedural analgesia. At termination, mice were euthanized by CO_2_ inhalation followed by cervical dislocation.

### Gene-Targeted Mice

*Sulf1^−/−^ and Sulf2^−/−^* mice (9–12 weeks) used in this study were described previously [37], [60]. The mice were maintained on the C57Bl/6×129Sv/Ola genetic background by heterozygous mating. *Sulf1^−/−^/Sulf2^−/−^* mice were generated by crossing *Sulf1^+/−^/Sulf2^+/−^* mice with *Sulf1^+/−^/Sulf2^+/−^* mice or by mating between *Sulf1^+/−^/Sulf2^−/−^* mice.

### Corneal wounding

All experiments were performed in strict accordance with the recommendations in the Guide for the Care and Use of Laboratory Animals of the National Institutes of Health and in strict accordance with the ARVO Statement for the Use of Animals in Ophthalmic and Vision Research. The study was approved by the Institutional Animal Care and Use Committee of University of California of San Francisco (IACUC) with approval number AN085643. Mice were maintained under pathogen-free conditions in the UCSF barrier facility. Mice were anesthetized by using 125 mg/kg 2,2,2-tribromoethanol (Avertin; Sigma-Aldrich, St. Louis, MO) intraperitoneally and by topical 0.5% Proparacaine (Akorn, Buffalo Grove, IL). After wounding, topical 0.5% Proparacaine was placed again on the cornea for post-procedural analgesia. At termination, mice were euthanized by CO_2_ inhalation followed by cervical dislocation. For the wound healing studies, wounds were generated by removing a circular region (∼1 mm diameter) of the central corneal epithelium with an AlgerBrush II (The Alger Company, Inc.). The defect was monitored by sodium fluorescein staining (0.1% in PBS) immediately after wounding and at later times. Fluorescence was imaged (Fluo III Leica dissecting microscope, Nikon CCD Camera) and two-dimensional projections of relative wound area were quantified using ImageJ software. Healing was quantified as per cent of the wound area remaining at the end of the assay relative to the original wound area.

### Cells and cell culture

Primary cultures of mouse corneal epithelial (MCE) cells were established as described [34] under conditions that promoted epithelial differentiation [73]. Cells were seeded (2 corneas/well) into a 24- or 48-well plate coated with a placenta-derived extracellular matrix (ECM) (Celprogen, San Pedro, CA) and grown to confluency for 5–7 days in supplementary hormonal epithelial medium (SHEM). SHEM consisted of equal volume HEPES-buffered DMEM and F12 medium, containing 10 ng/mL mouse-derived EGF, 5 µg/mL insulin, 5 µg/mL transferrin, 5 ng/mL sodium selenite, 0.5 µg/mL hydrocortisone, 0.1 µg/mL cholera toxin A subunit (all from Sigma-Aldrich), 5% FBS, 50 µg/mL gentamicin, and 1.25 µg/mL amphotericin B. THCE cells are SV40-immortalized human corneal epithelial cells [74] and were cultured in supplemented hormone epithelial medium (SHEM) [75].

### Constructs, lentiviral production and transfection

To achieve Sulf-2 knockdown in THCE cells, lentiviral transduction of *SULF2* shRNAs was employed [24], [25], [33]. For transient SULF overexpression, cells were transfected with cDNAs [16] using Fugene (Roche Diagnostics, IN) according to manufacturer's instructions. Super8XTopFlash, Super8XFopFlash and RL-CMV were provided by Dr. Randy Moon, University of Washington.

### Cell migration assay

THCE cells transduced with control shRNA or *SULF2* shRNA were plated (1×10^5^ cells) onto uncoated plastic 24-well plates and grown to confluency. MCE cells were grown on ECM-coated plates. Scratch wounds were made with a 10 µL pipette tip. In indicated experiments, Wnt pathway inhibitors sFRP and Dkk1 (R&D Systems, Minnesota, MN, Cat # 1384-SF-025, #5439-DK-010) were added to the cultures at recommended concentrations (1.2 µg/ml DKK1 and 5 µg/ml sFRP-1). Cultures were photographed at various time points until gap closure occurred. For time-lapse study, bright-field time-lapse movies were collected on a Zeiss Axiovert S-100 microscope by using a 10× A-Plan objective lens, a Ludl shutter, a Cohu CCD camera, and a Ludl x-y-z motorized stage. Temperature was held at 37°C and CO_2_ was held at 5% by using a CTI Controller 3700 and Temperature Control 37.2 combination. Images were acquired every 15 min by using MetaMorph (Molecular Devices, Inc., Sunnyvale CA). Wound area was measured with ImageJ, and cell migration was determined either as per cent of the wound area relative to the original wound area or as mean velocity of cells at the wound edge.

### RNA isolation and PCR

To determine the level of *SULF1 and SULF2* mRNA, RT-PCR was applied to cDNA prepared from either MCE, PCE or THCE. Total RNAs were extracted from cells using Trizol (Invitrogen) and cDNA was generated using Superscript II Reverse Transcriptase (Invitrogen). cDNA products were amplified using the following PCR primers: *MSULF1* (208 bp): F 5″-CACGTACTTCCCAGTGAGCA-3′ R 5′-TGCACGTACTTCCCAAGTGAG-3′ (Tannealing = 64.5°C). *MSULF2* (224 bp) F 5′- AATGCCCAGGAGGAGAAC-3′ R 5′-CTCTGGCCGTCATACTTG (T annealing = 58.5°C). The conditions for denaturation, and extension of the template cDNA were 94°C for 30 s; 72°C for 1 min for 35 cycles. The PCR products were separated by electrophoresis on 1.5% agarose gels and visualized with ethidium bromide. For measurements of *HSULF* levels, the following primers were used: *HSULF1* (371 bp product) F 5′-CTCACAGTCCGGCAGAGCACGCGG AAC, R 5′-CACGGCGTTGCTGCTATCTGC CAG CAT CC 3′ (Tannealing = 64C°). *HSULF2* (314 bp): F 5′-GAA AAG AGG CAG ATTCACGTCGTTTCCAG R 5′- ATCTGGTGCTTCTTTTGGGATGCGGGAG -3′ (T annealing = 64°C).

### Staining and Western blotting

For SULF1 expression in whole mounts, eyes of 8-week old male C57/BL6 mice were enucleated 4–72 hrs after corneal wounding. Corneas were prepared as described [76]. Briefly, the corneas were dissected to remove the lens, iris, and retina and five-seven incisions were made to flatten the cornea, followed by a fixation in 4% paraformaldehyde in PBS for 2 hrs. The tissue was incubated with 5 µg/ml R1.1 or with 5 µg/ml mAb 1A4 (Novus Biologicals, Littleton, CO) for SULF1, or either rabbit anti-DNP IgG primary antibody (Invitrogen, Carlsbad, CA) or mouse IgM as their respected controls at the same concentrations for 2–3 days at 4°C in 0.2% BSA/5% goat serum/0.3% Triton-X100 in PBS. Reaction with the primary antibody was followed by the sequential addition of biotinylated goat-anti rabbit or goat-anti mouse (Jackson ImmunoResearch Laboratories, West Grove, PA) for 2–3 hrs at RT, and by streptavidin-Cy3 conjugated antibody (Jackson ImmunoResearch Laboratories) for 2 hrs. Tissues were also stained with DAPI (Roche Molecular Biochemicals, Indianapolis, IN) for 15 min. The tissue was post-fixed in 4% paraformaldehyde and mounted on the slide epithelial side up. Whole mounts were imaged by laser scanning confocal (Leica SP2) or by fluorescence microscopy (Nikon Optiphot).

Detergent cell lysates and conditioned medium (CM) of corneal epithelial cells were prepared as described [33]. Lysates and CM at equalized protein concentrations based on the BCA™ Protein Assay (Pierce, Rockford, IL) were subjected to conventional SDS-PAGE on 7.5% gels (BioRad), blotted to ProBlott™ (Applied Biosystems). Immunoblotting was performed with 1 µg/ml mouse SULF1 mAb 1A4 which recognizes the C-terminal 50-kDa subunit (Novus Biologicals, Littleton, CO), 3 µg/ml G1.6 for SULF1 (see **Figure S3B**) and 1 µg/ml R2.3 and R2.1 for SULF2, both of which detect the N-terminal 75-kDa subunit [32]. Secondary antibodies were 1∶3000 HRP-goat anti-mouse IgM or HRP-swine-anti-goat IgG isotype for SULF1 and HRP-goat anti-mouse IgG for SULF2 (Jackson ImmunoResearch). Signals were detected with ECL (BioRad Laboratories, Hercules, CA).

To determine the effects of SULF2 knockdown on the sulfation status (6OS) of HSPGs of THCE cells, the phage display antibody RB4CD12 (alternative designation is HS3A8) [35], [36] was used in conjunction with a Cy3-conjugated mouse anti-VSV secondary antibody (Sigma, St Louis, MD). Cells were analyzed by fluorescent microscopy and by flow cytometry.

### Wnt Signaling Reporter Assay

To examine the effect of the SULF on the Wnt/ß-catenin signaling, THCE cells transduced with control shRNA or *SULF2* shRNA were seeded onto a 24-well plate and at ∼50–60% confluency transfected with either Super8XTopFlash or Super8XFopFlash at 0.5 µg/well and RL-CMV at 0.1 µg/well control plasmid, at 3∶1 Fugene∶DNA ratio. Cells were analyzed 48 hrs post-transfection using the Dual-Luciferase Reporter Assay System (Promega, Madison, WI). Luciferase measurements were carried out in a TD-20/20 luminometer. Super8xTOPflash and andSuper8xFOPflash signal was normalized to the Renilla signal, and the TOP/FOP ratio was calculated for each cell type. The data are presented as individual ratio and as per cent of shRNA SULF2 relative to the shRNA control.

## Supporting Information

Figure S1 **Morphology of** ***Sulf1^−/−^/Sulf2*** **^−/−^ cornea.** H&E staining of paraffin-embedded corneas of the wild type (WT) and *Sulf1* ^−/−^ *Sulf2* ^−/−^ mice. Both corneas show no apparent difference in thickness, with 3–4 layers of epithelium, well-organized stoma with similar number of keratocytes (arrowhead) and a single layer of endothelial cells.
(TIF)

Figure S2 ***Sulf*** **mRNA expression in wounded and contralateral non-wounded cornea.** Real-time PCR was performed on cDNA prepared from needle-scratched corneas and contralateral control corneas 24 hrs after injury (pools of 15 corneas each). *MSulf1 and MSulf2* mRNA expression were normalized relative to β-*actin* expression (HKG). Means+SEMs are shown, N = 2. *Sulf1* expression increased in the injured corneas. *p = 0.05, Student t-test. *Sulf2* expression did not significantly change with injury.
(TIF)

Figure S3 **Characterization of SULF1 antibodies.** **A:** R1.1 and **B:** G1.6. Conditioned medium from HEK 293T cells (parental) or cells transfected with pcDNA *MSulf1* or pcDNA *MSulf2* was collected, concentrated, and analyzed by ELISA in which the CMs were captured onto plastic and reacted with the indicated IgG, an HRP-conjugated secondary antibody plus substrate for color generation (405 nm). X-axis indicates CM dilution and Y-axis indicates reactivity of the specific antibody and isotype control IgG.
(TIF)

Figure S4 **Staining of injured mouse cornea with alternative anti SULF1 antibody.** Whole mounts of 18 hr post-wounded corneas were stained with either anti SULF1 antibody (mA14) or mouse IgM (isotype control) and imaged by laser scanning microscopy at 20×. SULF1 was concentrated around the edge of the wound in the superficial cells. No signal was present with the control antibody. The dotted white lines approximate the margin of the wounds. Scale bar: 100 µm.
(TIF)

Figure S5 **The effect of SULF2 shRNA on proliferation of THCE cells.** THCE cells were transduced with mock shRNA (Cnt shRNA) or *HSULF2* shRNA1. At 80% confluency, the cells were incubated with BrdU for 2 hrs and analyzed for incorporation. Representative flow cytometry dot plots are shown for one of three sample pairs. There was no statistical difference between the two groups, Student t-test.
(TIF)

Methods S1 (DOC)

Movie S1 **Time-lapse video of WT MCE cell migration in a scratch-wound assay.**
(MP4)

Movie S2 **Time-lapse video of** ***Sulf1^−/−^*** **MCE cell migration in a scratch-wound assay.**
(MP4)

Movie S3 **Time-lapse video of** ***Sulf2^−/−^*** **MCE cell migration in a scratch-wound assay.**
(MP4)

Movie S4 **Time-lapse video of Cnt shRNA THCE migration in a scratch-wound assay.**
(MP4)

Movie S5 **Time-lapse video of** ***HSULF2*** **shRNA1 THCE migration in a scratch-wound assay.**
(MP4)
