# Supplementary figures and images for: The SULFs, Extracellular Sulfatases for Heparan Sulfate, Promote the Migration of Corneal Epithelial Cells during Wound Repair
Source: PLoS One. 2013 Aug 8;8(8):e69642. doi: 10.1371/journal.pone.0069642 (PMC3738537; doi:10.1371/journal.pone.0069642)

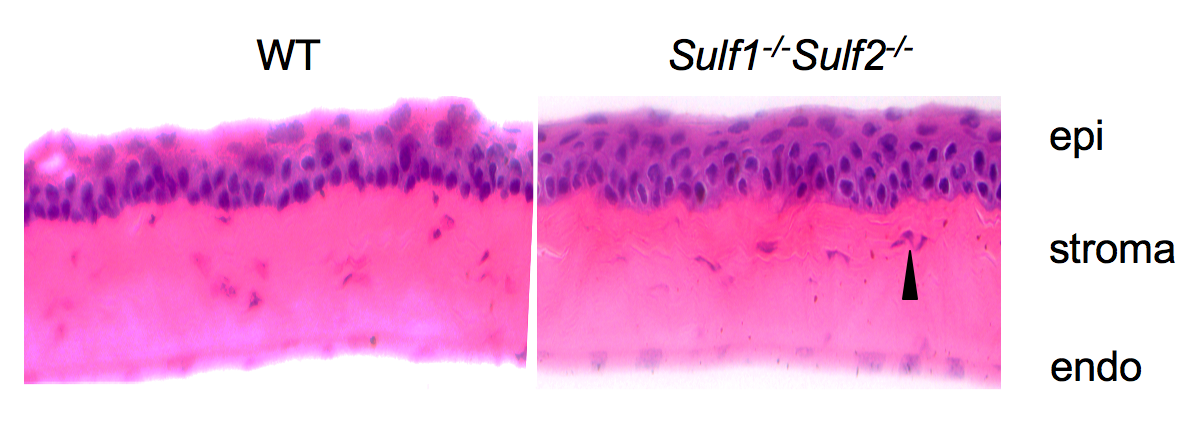

Supplement: Figure S1 — Morphology of Sulf1−/−/Sulf2 −/− cornea. H&E staining of paraffin-embedded corneas of the wild type (WT) and Sulf1 −/− Sulf2 −/− mice. Both corneas show no apparent difference in thickness, with 3–4 layers of epithelium, well-organized stoma with similar number of keratocytes (arrowhead) and a single layer of endothelial cells. (TIF) [file pone.0069642.s001.tif]

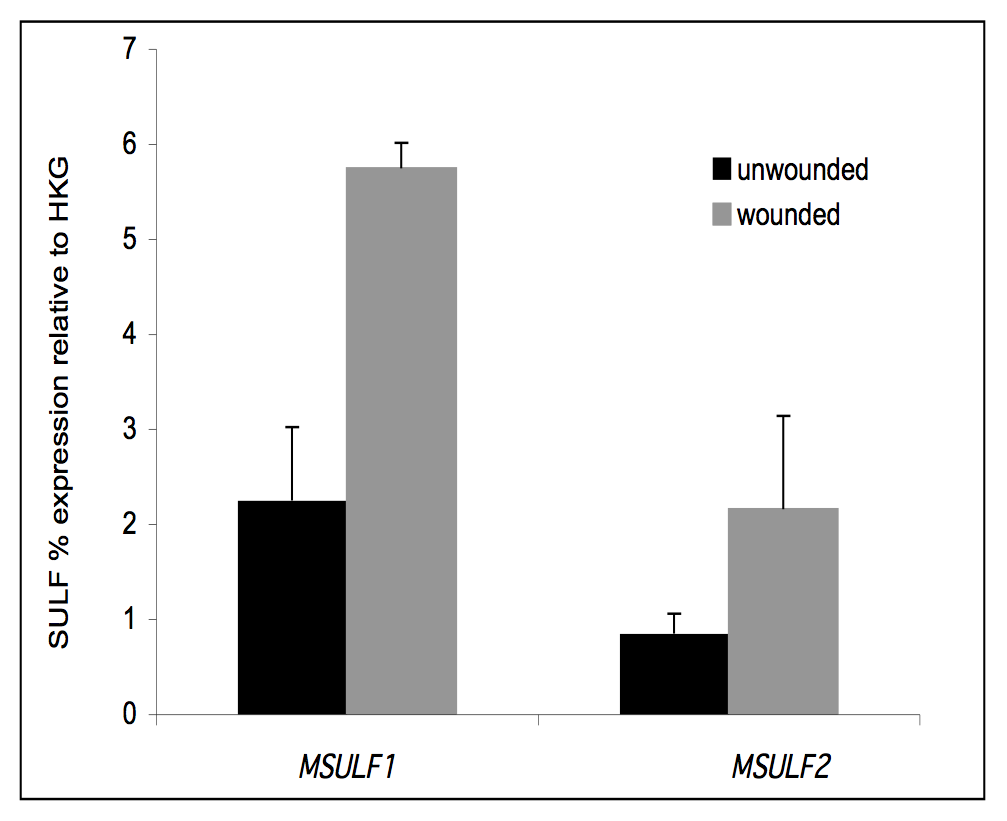

Supplement: Figure S2 — Sulf mRNA expression in wounded and contralateral non-wounded cornea. Real-time PCR was performed on cDNA prepared from needle-scratched corneas and contralateral control corneas 24 hrs after injury (pools of 15 corneas each). MSulf1 and MSulf2 mRNA expression were normalized relative to β-actin expression (HKG). Means+SEMs are shown, N = 2. Sulf1 expression increased in the injured corneas. *p = 0.05, Student t-test. Sulf2 expression did not significantly change with injury. (TIF) [file pone.0069642.s002.tif]

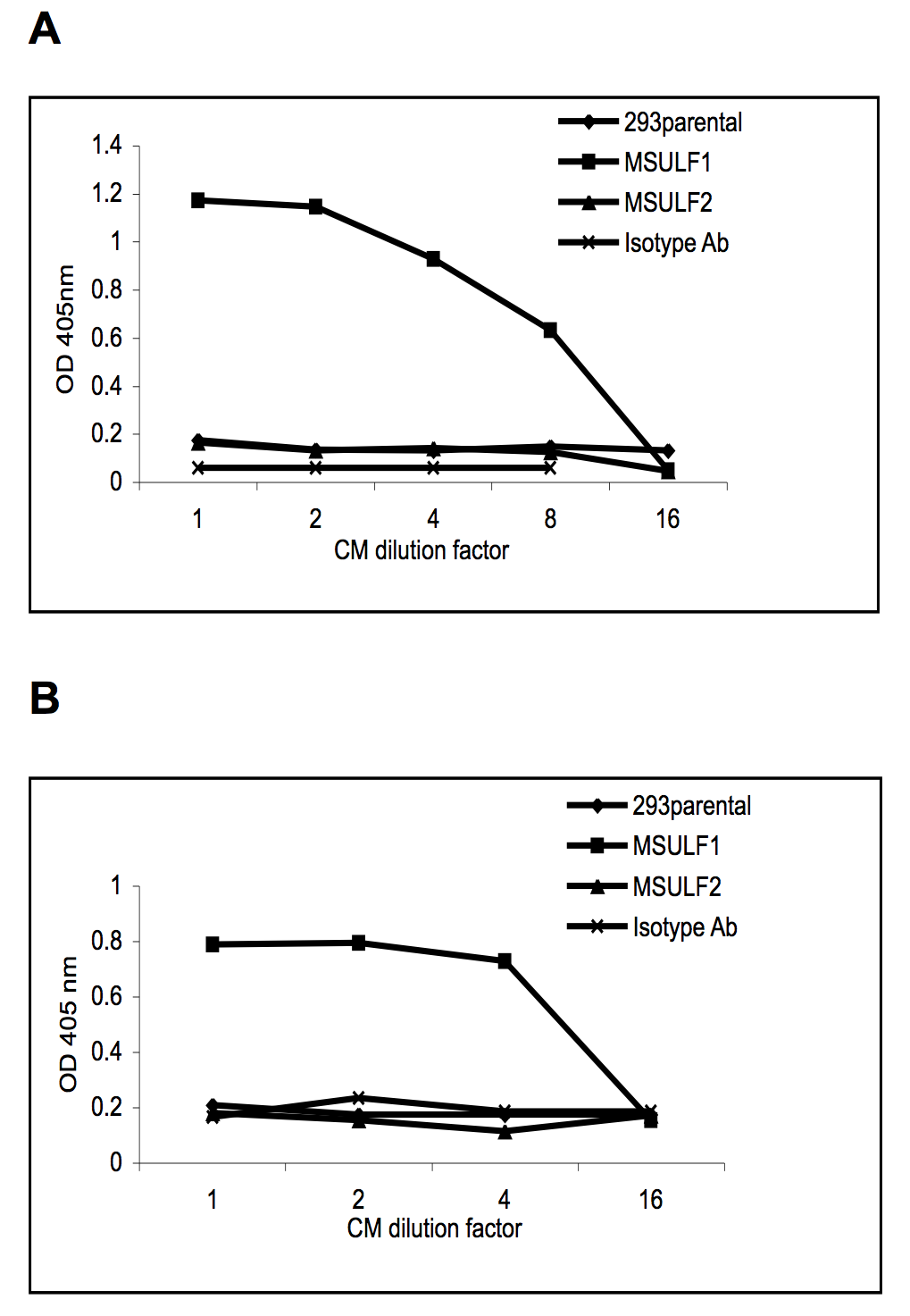

Supplement: Figure S3 — Characterization of SULF1 antibodies. A: R1.1 and B: G1.6. Conditioned medium from HEK 293T cells (parental) or cells transfected with pcDNA MSulf1 or pcDNA MSulf2 was collected, concentrated, and analyzed by ELISA in which the CMs were captured onto plastic and reacted with the indicated IgG, an HRP-conjugated secondary antibody plus substrate for color generation (405 nm). X-axis indicates CM dilution and Y-axis indicates reactivity of the specific antibody and isotype control IgG. (TIF) [file pone.0069642.s003.tif]

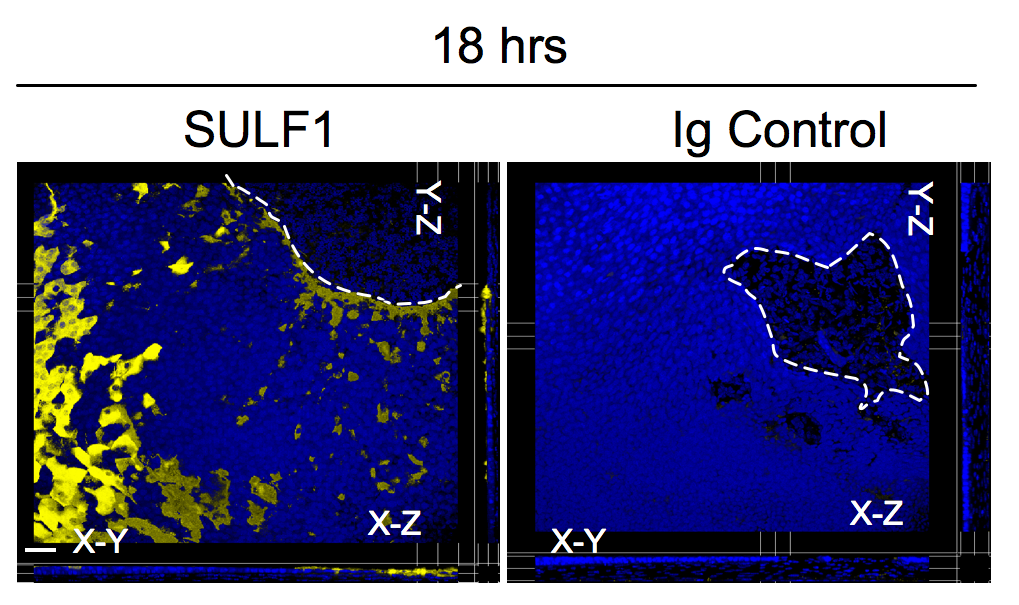

Supplement: Figure S4 — Staining of injured mouse cornea with alternative anti SULF1 antibody. Whole mounts of 18 hr post-wounded corneas were stained with either anti SULF1 antibody (mA14) or mouse IgM (isotype control) and imaged by laser scanning microscopy at 20×. SULF1 was concentrated around the edge of the wound in the superficial cells. No signal was present with the control antibody. The dotted white lines approximate the margin of the wounds. Scale bar: 100 µm. (TIF) [file pone.0069642.s004.tif]

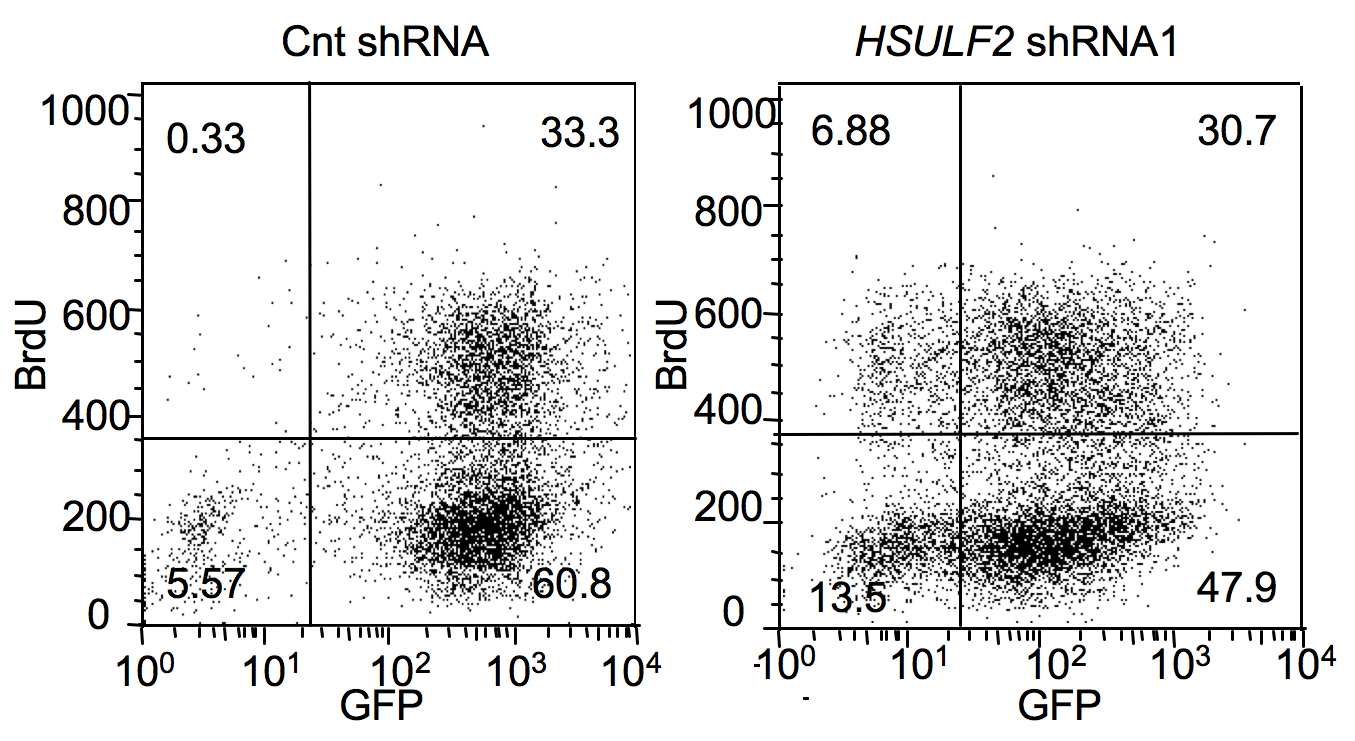

Supplement: Figure S5 — The effect of SULF2 shRNA on proliferation of THCE cells. THCE cells were transduced with mock shRNA (Cnt shRNA) or HSULF2 shRNA1. At 80% confluency, the cells were incubated with BrdU for 2 hrs and analyzed for incorporation. Representative flow cytometry dot plots are shown for one of three sample pairs. There was no statistical difference between the two groups, Student t-test. (TIF) [file pone.0069642.s005.tif]
