# Supplementary material for: The SULFs, Extracellular Sulfatases for Heparan Sulfate, Promote the Migration of Corneal Epithelial Cells during Wound Repair
Source: PLoS One. 2013 Aug 8;8(8):e69642. doi: 10.1371/journal.pone.0069642 (PMC3738537; doi:10.1371/journal.pone.0069642)
Supplement: Methods S1 — (DOC) [file pone.0069642.s006.doc]

**Supporting Information Methods**

***Measurement of Sulf* transcripts by real time PCR**

For the initial RNA expression study, 15 experimental corneas of Balb/C mice were wounded by 5 vertical and 5 horizontal scratches made with a 26-gauge needle, while the control contralateral corneas were left unwounded. Corneas were collected 24 hrs after wounding and processed for qRT-PCR with SYBR Green (Applied Biosystems, Carlsbad, CA). cDNA products were amplified using the following PCR primers: *MSULF1* F: 5’-AACCTG AGGAGTGTGGCTGT-3’, R: 5’-TGAAGGGGTGAAGGTGACTC-3’. MS2 F: 5’-CAA CTA TGC ACC CAA CCC AGA-3’

R: 5’-CCA GCA TGT CAT AGA TCG TC-3’. C57/Bl6MGAPDH F: 5’-tgtgtccgtcgtggatctga-3’, R: 5’-ACCACCTTCTTGATGTCATCATACTT-3’. The conditions for denaturing, annealing and extension of the template cDNA were: 94°C for 30 sec, 55°C for 30 sec and 72°C for 1 min for 35 cycles.

**Preparation of Anti–SULF1 Polyclonal Antibodies**

The mature form of each SULF consists of a disulfide-linked heterodimer of 75 and 50-kD subunits, which are formed by proteolytic processing of a 125 kDA precursor protein [1]. Polyclonal antibodies were produced by ProSci (Poway, CA). Rabbits were immunized with a KLH conjugate of a peptide derived from the predicted sequence of HSULF1 at the amino-terminus (CSTVRSPRFRGRIQQERKN) of the mature protein [1], where C denotes the cysteine residue added for coupling). The peptide is highly homologous to the corresponding region in MSULF1 (18/19 residues identical), but lacks homology to the corresponding regions in HSULF2 and MSULF2. The antibody, termed R1.1, was affinity purified on a column comprised of the peptide coupled to SulfoLink Coupling Gel (Pierce, Rockford, IL). For the production of G1.6, a goat was immunized with a KLH conjugate of CKKEESSKNIQQSNH, which is present in the hydrophilic domain of HSULF1 (75 kDa subunit), where C denotes the cysteine residue added for coupling. The peptide is identical in 13/14 residues to MSULF1 but lacks homology to SULF2. The antibody was purified by affinity chromatography as above.

**ELISA for SULF1 detection**

As a source of SULF protein, we used HEK 293T cells transiently transfected with either pcDNA *MSulf1* or pcDNA *MSulf2* [16] and the parental HEK 293T cells (293T) as the control. SULF1 and SULF2 expressing HEK 293 cells were grown in Dulbecco's modified Eagle's (DMEM) medium supplemented with 10% fetal bovine serum. OptiMEM (Invitrogen) replaced standard growth medium to obtain conditioned medium (CM). CM was collected from a 10 cm dish after 72 hr post-transfection, was clarified by centrifugation at 5,000 RPM for 5 min and concentrated 10x on a Centricon 30 microconcentrator (Millipore Corp.). A 96-well plate (Immulon 2HB, Dynex Laboratories) was coated at 4°C overnight with 100 ng of heparin-BSA [2]. The wells were washed with PBS containing 0.1% Tween-20 (PBS-T) and then blocked with 3% BSA in PBS at RT for 2 hrs. The wells were rinsed and incubated with serial dilutions of CM in 100 µL at RT for 2 hrs. Washed wells were incubated with 100 µL/well of SULF1 (R1.1 or G1.6) or isotype control (rabbit IgG or goat IgG, 5 µg/mL) in 1% BSA/PBS at RT for 2 hrs, followed by washes and by an incubation with 100 µL/well of secondary HRP-conjugated antibody (goat anti-rabbit for R1.1 or swine-anti-goat for G1.6) at RT for 1 hour. The wells were finally exposed to ABS substrate (Vector Laboratories, Burlingame, CA) at RT for 5-30 min. OD 405 nm was read on a microplate reader (Bio-Rad, Hercules, CA).

**Histochemistry**

To examine corneal gross morphology, corneas of wild type and *Sulf1^-/-^Sulf2*^-/-^ mice were fixed with 4% PFA, dehydrated sequentially in ethanol, embedded in paraffin for 5 micron sectioning. Sections were de-paraffinized, re-hydrated and H&E stained.

**Cell Proliferation Assay**

To determine the cell proliferation rate of mock- and SULF2 knockdown THCE, cells were grown to ~80% confluency and pulsed with BrdU (10 μl of 1 mM BrdU per 1 ml of medium) for 2 hrs, after which the cells were stained using the FITC BrdU Flow kit (BD Biosciences) and quantified by flow cytometry (Becton-Dickinson, FACSscan), as per the instructions.

**References**

1. Tang R, Rosen SD (2009) Functional consequences of the subdomain organization of the sulfs. J Biol Chem 284: 21505-21514.

2. Uchimura K, Morimoto-Tomita M, Rosen SD (2006) Measuring the activities of the Sulfs: two novel heparin/heparan sulfate endosulfatases. Methods Enzymol 416: 243-253.
